# Methods and novel technology for microRNA quantification in colorectal cancer screening

**DOI:** 10.1186/s13148-017-0420-9

**Published:** 2017-10-24

**Authors:** Laura Moody, Hongshan He, Yuan-Xiang Pan, Hong Chen

**Affiliations:** 1 Division of Nutritional Sciences, University of Illinois at Urbana-Champaign, 472 Bevier Hall, MC-182, 905 South Goodwin Avenue, Urbana, IL 61801 USA; 2 Department of Food Science and Human Nutrition, University of Illinois at Urbana-Champaign, 472 Bevier Hall, MC-182, 905 South Goodwin Avenue, Urbana, IL 61801 USA; 3 Illinois Informatics Institute, University of Illinois at Urbana-Champaign, Urbana, IL 61801 USA; 4 Department of Chemistry, Eastern Illinois University, Charleston, IL 62910 USA

**Keywords:** Colorectal cancer, Isothermal, Near-infrared (NIR), miRNA, Diagnosis, Optical imaging, Ytterbium

## Abstract

The screening and diagnosis of colorectal cancer (CRC) currently relies heavily on invasive endoscopic techniques as well as imaging and antigen detection tools. More accessible and reliable biomarkers are necessary for early detection in order to expedite treatment and improve patient outcomes. Recent studies have indicated that levels of specific microRNA (miRNA) are altered in CRC; however, measuring miRNA in biological samples has proven difficult, given the complicated and lengthy PCR-based procedures used by most laboratories. In this manuscript, we examine the potential of miRNA as CRC biomarkers, summarize the methods that have commonly been employed to quantify miRNA, and focus on novel strategies that can improve or replace existing technology for feasible implementation in a clinical setting. These include isothermal amplification techniques that can potentially eliminate the need for specialized thermocycling equipment. Additionally, we propose the use of near-infrared (NIR) probes which can minimize autofluorescence and photobleaching and streamline quantification without tedious sample processing. We suggest that novel miRNA quantification tools will be necessary to encourage new discoveries and facilitate their translation to clinical practice.

## Background

Colorectal cancer (CRC) is one of the most common types of human cancers with high cancer-related morbidity and mortality rates. Low survival rates among metastatic CRC patients highlight the need for early detection in order to keep the tumor localized and dramatically improve prognosis [1]. In the early stages of the disease, CRC presents with the formation of benign polyps, or adenomas, in the colorectal mucosa. If found early, surgical intervention can remove adenomas before they become cancerous, but if untreated, adenomas can advance into invasive tumors and metastasize to the lymph nodes and other organs. Patients with cancer metastases only have a 10% 5-year survival rate, but intervention during the early stages of the disease improves the survival rate to 90% [2]. The progression from adenoma to cancer can take 10–15 years, allowing for ample time to detect colon abnormalities [3]. However, current screening tools are primarily invasive and require specialized equipment. Thus, identification of effective noninvasive screening measures has tremendous potential to increase survivorship.

### Current CRC screening tools: a brief overview

Currently, endoscopic procedures are the primary means of colorectal cancer screening. Colonoscopy is an invasive procedure that has been used for decades to visualize the entire colon and is recommended every 10 years for individuals over the age of 50 [4]. Numerous studies have established colonoscopy as a valuable screening tool that decreases CRC incidence up to 76% and lowers mortality by up to 65% [5–7]. The sensitivity of colonoscopies for colorectal adenomas ranges from 75 to 93% [4] while specificity approaches 100% [8]. A less invasive but less thorough alternative to colonoscopy is sigmoidoscopy in which only the lower part of the colon is examined. Sigmoidoscopy offers a sensitivity of 77 to 84% and a specificity of around 84% [9, 10]. Several studies have shown that sigmoidoscopy also decreases rates of CRC onset [11, 12]. One group showed that in patients screened with sigmoidoscopy, CRC development was reduced by 23% compared to unscreened controls and mortality was decreased by 31% [13]. While compliance in this study was high (71%), other reports indicate that only 50% of the general population adheres to screening recommendations [14, 15]. Because of its invasive nature, patients often report discomfort or pain during the procedure, which may prevent full compliance. Although rare, endoscopy also poses a risk for complications such as intestinal perforation and bleeding (Table 1) [4].

**Table 1:** Current CRC screening methods

Tool	Blood or stool	Sensitivity (%)	Specificity (%)	Advantages	Limitations	Citation
Colonoscopy	Invasive	75–93	100	• Well validated and widely accepted • High sensitivity and specificity	• Requires expertise to perform/interpret • Invasive • Low compliance • Risk of intestinal perforation and bleeding	[4, 8]
Sigmoidoscopy	Invasive	77–84	84	• Typically does not require sedation • Less extensive bowel preparation	• Requires expertise to perform/interpret • Invasive • Risk of intestinal perforation and bleeding • Not as thorough as colonoscopy	[9, 10]
Fecal occult blood test (FOBT)	Stool	50	91–98	• Inexpensive • Can be performed at home	• Low sensitivity • Requires repeated testing	[18, 19]
Fecal immunochemical test (FIT)	Stool	93	90	• Inexpensive • Can be performed at home • High sensitivity	• Not as sensitive to colorectal neoplasia	[19, 21]
Cologuard	Stool	92–98	90	• Inexpensive • Can be performed at home	• Not as sensitive to colorectal neoplasia	[22, 24]
Carcinoembryonic antigen (CEA)	Blood	74–80	70–95	• Easy to perform	• Cannot detect early stage CRC • No standardized cutoff values	[27, 29]
Epi proColon	Blood	66–68	91	• Easy to perform	• Low sensitivity	[31, 32]

Several non-invasive alternatives to endoscopy have been proposed, but all are limited in sensitivity and specificity. The guaiac-based fecal occult blood test (FOBT) was developed to measure fecal blood in an inexpensive, noninvasive manner. Although the FOBT has been shown to reduce incidence and mortality of CRC [2, 5], the test is hindered by its insensitivity. False positives are common due to usage of mediations such as nonsteroidal anti-inflammatory drugs as well as dietary intake of red meat [16, 17]. A single FOBT has a sensitivity of only around 50% [18, 19], and while repeated testing can boost sensitivity, patients may be less willing to attend multiple appointments [20]. The fecal immunochemical test (FIT) is an antibody-based measurement of hemoglobin protein in stool. One study found that the FOBT provided a specificity of only 78% while the FIT offered a specificity of over 90% [19]. The FIT also has a higher average sensitivity for CRC of 93%, but has only a 48% sensitivity for advanced neoplasia, suggesting that it may not be optimal for identifying individuals at risk for CRC [21].

In 2014, the multitarget DNA test Cologuard® was FDA-approved and made commercially available in the USA. In addition to fecal hemoglobin, Cologuard® assays DNA methylation in the bone morphogenetic protein 3 (BMP3) and NDRG family member 4 (NDRG4) promoters and mutations in the KRAS proto-oncogene (KRAS) gene. The assay was reported to detect CRC with a sensitivity of 92% and premalignant lesions with a sensitivity of 42% [22]. In a screening of over 400 asymptomatic adults, methylation of BMP3 showed greater specificity for polyp detection than the FIT [23]. Another study in over 1000 subjects found that Cologuard® detected CRC with 90% specificity and 98% sensitivity [24]. It was further noted that Cologuard® could detect precancerous lesions with a sensitivity of 57% for precursors ≥ 1 cm and 83% for precursors > 3 cm. This evidence suggests that fecal genetic markers can provide a viable means of cancer detection.

Like fecal screening, blood-based CRC biomarkers are not common and are limited in their ability to detect at-risk individuals. The carcinoembryonic antigen (CEA) is a glycoprotein associated with various cancers as well as gastrointestinal and liver diseases. CEA is a blood-based biomarker for CRC detection, although the sensitivity and specificity for early-stage CRC detection are relatively low [25, 26]. Indeed, the sensitivity and specificity of CEA for detecting CRC up to 1 year before clinical presentation is only 57.5 and 81%, respectively [27]. Elevated CEA levels in serum are correlated with higher CRC mortality rates [28]; however, early stages of CRC do not necessarily display high levels of CEA, and therefore, CEA is not widely accepted as a reliable diagnostic measure [29, 30].

In 2016, the first blood-based colorectal cancer screening test was approved by the FDA. Epi proColon® measures methylated Septin9 (SEPT9) and was demonstrated to have a sensitivity of 68% [31]. Meta-analysis of 9870 subjects showed that methylated SEPT9 had a 66% sensitivity and 91% specificity for CRC [32]. The analysis suggests that these values are comparable to those provided by the FOBT, and thus, the authors propose Epi proColon® as a complementary screening tool. Although not as sensitive as endoscopy, Epi proColon® highlights the potential of blood-based epigenetic assays in CRC screening.

### miRNA in CRC

miRNAs are small RNAs that regulate the expression of mRNA expression by binding to the 3′ untranslated region (UTR) and altering ribosomal interactions, decapping or deadenylating the mRNA, or recruiting the RNA-induced silencing complex (RISC) [33]. miRNAs are involved in human carcinogenesis and alter expression of oncogenes and tumor suppressor genes as well as disrupt cellular functions such as stress response and apoptosis [34]. Tumor growth and metastasis in various cancer types can be mediated by dysfunctional regulation by miRNA [35–38].

Not only do tumors display aberrant miRNA expression, but biological fluids also show altered miRNA levels in cancer. Recent evidence suggests that miRNA may be excreted into circulation via exosomes and microvesicles or bound to proteins such as Argonaute or high-density lipoprotein (HDL) [39]. Indeed, several studies have demonstrated unique circulating miRNA profiles due to a wide variety of diseases, dietary patterns, and lifestyle factors [40–42]. Unlike in other cancers, fecal samples may potentially be used for patient screening in CRC. miRNA from tumor cells that slough off the lumen wall can be isolated in stool for quantification. Table 2 highlights the utility of noninvasive miRNA biomarkers for detecting CRC and identifying at-risk individuals.

**Table 2:** Potential miRNA biomarkers for CRC screening

miRNA	Blood or stool	Sensitivity (%)	Specificity (%)	Citation
miR-92a	Blood and stool	72–79 (blood) 72 (stool)	59–69 (blood) 73 (stool)	[60, 66]
miR-20a	Blood and stool	46 (blood) 55 (stool)	73 (blood) 82 (stool)	[46, 67]
miR-21	Blood and stool	62–85 (blood) 56 (stool)	79–88 (blood) 73 (stool)	[52, 55, 66]
miR-221	Blood and stool	86 (blood) 62 (stool)	41 (blood) 74 (stool)	[51, 68]
miR-18a	Stool	61	69	[68]
miR-135b	Stool	78	68	[66]
miR-144*	Stool	74	87	[67]
miR-199a-3p	Blood	48	75	[50]
miR-155	Blood	58	95	[48]
miR-183	Blood	74	89	[56]
miR-29a	Blood	53–65	85–93	[57]
miR-29b	Blood	61	73	[47]
miR-210	Blood	75	74	[53]
miR-196b	Blood	63	87	[54]
miR-139-3p	Blood	97	98	[49]
miR-622	Blood	88	64	[49]
miR-506	Blood	61	77	[72]
miR-4316	Blood	75	77	[72]

#### Circulating miRNA

Circulating miRNA may serve as a reflection of the underlying disease in CRC [43–45]. Several plasma miRNA have been shown to be dysregulated in CRC (Table 2) [46–57]. miR-92 was first reported as a possible noninvasive biomarker for CRC diagnosis in 2009 [58, 59]. Since then, a recent meta-analysis of over 500 colorectal cancer patients reported that miR-92a had a diagnostic sensitivity of 76% and sensitivity of specificity of 64% [60]. When several miRNAs (let-7g, miR-21, miR-92a, miR-181b, and miR-203) in serum were used as a biomarker profile panel for CRC diagnosis, sensitivity and specificity increased to 93 and 91%, respectively. The same serum samples showed only 35% sensitivity and 23% specificity for CRC when CEA and CA19-9 were used [61]. In addition to distinguishing normal individuals from cancer patients, circulating miRNA profiles have been shown to differ between healthy controls and patients with pre-cancerous adenomas [62]. High-throughput sequencing has facilitated the discovery of many more circulating miRNA that are differentially expressed in CRC [63, 64]; however, no blood-based miRNA tests are currently being used to screen for CRC.

#### Fecal miRNA

The FOBT and FIT are the primary stool-based screening tools for CRC, but the sensitivity and specificity are relatively low, especially at early pre-malignant time points [65]. Studies have reported that miR-92a [66], miR-20a [67], miR-21 [66], miR-221 [68], miR-18a [68], and miR-144* [69] are differentially expressed in CRC patients in comparison to healthy volunteers. These studies report sensitivities ranging from 55 to 74% and specificities ranging from 68 to 87%. Table 2 demonstrates the efficiency of each fecal miRNA as a CRC biomarker. A majority of the studies investigating fecal miRNA are exploratory and are conducted on relatively small sample sizes. As more significant fecal miRNA biomarkers surface, adequate measurement tools will become more important to ensure reliable quantification.

### Conventional miRNA quantification tools

While basic science and clinical trials have demonstrated a role for miRNA in colorectal cancer, the feasibility of clinical implementation remains questionable. Not only are sensitivity and reproducibility important characteristics of cancer biomarkers, but time, cost, and complexity of sample processing are crucial in facilitating the transition from the laboratory to the clinic. Currently, quantitative real-time PCR (qPCR), microarray, and next-generation sequencing (NGS) are the most commonly used techniques for quantifying miRNA. While such procedures are routinely used in the laboratory, not one is completely ideal for rapid, reproducible miRNA detection. Here, we outline the chemistry involved in conventional quantification tools. We draw attention to the advantages of each platform and discuss areas where each falls short (Table 3).

**Table 3:** miRNA quantification technology

Technology	Advantages	Limitations	Citations
qPCR	• Current gold standard for sensitivity and specificity	• No genome-wide coverage	[74–76]
Microarray	• Commercially available reagents • Genome-wide coverage	• Specific probes • Specialized equipment • Lack of reproducibility between platforms • Difficult data normalization	[81–87]
NGS	• Genome-wide coverage • Multiple samples may be run in parallel • Promotes novel miRNA discovery • Can detect polymorphisms	• Complicated, non-standardized data analysis	[93–97]
Isothermal amplification	• No need for thermocycling equipment • Can improve existing qPCR, microarray, and NGS methods	• Disadvantages are technique-specific (see below)	[101]
• Exponential amplification	• High sensitivity	• May require a nicking enzyme, which complicates primer design	[102, 103]
• Rolling circle amplification	• 1 primer • Can be optimized for linear or exponential amplification	• Requires 2 enzymes (polymerase and ligase) • Initial denaturation not performed at room temperature	[105–109]
• Duplex-specific nuclease signal amplification	• High specificity	• Enzyme is not readily available	[110–113]
• Hybridization chain reaction	• No polymerase	• Linear amplification only	[114–120]
Near-infrared technology	• No autofluoresence • Minimal photobleaching • No tedious treatment of sample before or after the test	• Lanthanide probes are not yet commercially available and must be optimized	[132, 140–143]

#### qPCR

qPCR is a means of measuring single miRNAs and is currently the gold standard for miRNA quantification, and it has been routinely utilized for measuring miRNA in both blood [70–72] and stool [66, 69, 73] samples in order to explore miRNA for CRC screening. Typically, after miRNA is isolated, it is amplified with reverse transcription (RT)-PCR to make cDNA. The sequence of interest is then amplified and measured in real time using fluorescent probes. SYBR Green and Taqman chemistry are the two primary systems used for fluorescent detection. SYBR Green binds double-stranded DNA and fluoresces as the target sequence is amplified. This eliminates the need for separate probes for each miRNA of interest, but reduces the assay’s specificity, as all double-stranded DNA fragments produce fluorescence. Taqman technology relies on a labeled probe. During the qPCR reaction, the labeled probe binds to the target sequence. The primers anneal and are extended by Taq polymerase, which degrades the probe and releases the fluorophore. While Taqman provides superior miRNA detection sensitivity and specificity, labeled probes must be synthesized for every region of interest, which is less cost-effective when quantifying numerous genes.

miRNA differs from mRNA in that sequences are short (~ 22 nt) and are generally less abundant. Thus, qPCR procedures must avoid primer dimers and ensure a low detection threshold. Currently, two methods attempt to overcome these hurdles. The use of stem-loop RT primers has been shown to improve miRNA detection sensitivity and specificity over linear primers, potentially via spatial constraints, base-stacking, and increased thermal stability [74]. However, in order to enrich the assay for the miRNA of interest, this method requires separate RT primers for each miRNA. Alternatively, Exiqon has developed a locked nucleic acid (LNA) assay in which the RT-PCR reaction needs only to be performed once. The RT reaction is performed with a universal mature miRNA primer, and for qPCR, LNA primers may be used with SYBR Green for amplification. LNAs have a higher affinity to complementary bases. Therefore, shorter sequences may be synthesized to prevent primer overlap. While the LNA-based technique has efficiencies comparable to those of stem-loop primers, measurements are more variable, less efficient, and specificity may be an issue [75, 76].

#### Microarray

In order to identify novel biomarkers for CRC screening, microarray has been extensively used to quantify all human miRNA in both blood [77–79] and fecal samples [67, 80]. Microarray is a hybridization technique that utilizes DNA probes to quantify specific miRNAs. Much like in qPCR, isolated RNA is amplified by RT-PCR. Biotinylation and fragmentation are performed and the sample is incubated over several hours during which the sample hybridizes with probes that are fixed on the microarray surface. The microarray cartridge is then washed to remove non-specific binding. Finally, the plate is stained with streptavidin bound to a fluorophore. Streptavidin binds to the biotinylated sequences and the fluorophore can be excited by a laser.

Several microarray platforms have been adapted for miRNA quantification, including GeneChip (Affymetrix), miRCURY LNA (Exiqon), and SurePrint (Agilent). All platforms have designed probes specific for mature miRNA sequences, but major differences include hybridization and washing procedures as well as fluorescent dyes. Microarray technology can be used to measure multiple miRNA simultaneously, but several drawbacks limit their utility in the clinic. Specific probes, specialized equipment, and differences in hybridization procedures drive up cost and compromise reproducibility between platforms [81, 82]. Furthermore, data normalization is difficult and time-consuming, and no single method has been universally accepted to analyze microarray data [83–86]. miRNA poses an even greater challenge for normalization due to the small number of miRNA and weak expression levels [87].

#### Next-generation sequencing

Massively parallel next-generation sequencing (NGS) was first introduced in 2005 and has since enabled researchers to measure miRNA in a genome-wide fashion [88]. Several platforms are available from Life Technologies, Illumina, and others. The typical workflow involves RNA isolation, library preparation, sequencing, and data analysis. Library construction involves 5′ and 3′ adapter ligation and amplification. Adapters are platform-specific and provide a bar code that is recognized during amplification. Amplification has traditionally been performed using either emulsion PCR (emPCR) or bridge PCR (bPCR). The Sequencing by Oligonucleotide Ligation and Detection (SOLiD) platform developed by Life Technologies has employed emPCR. In emPCR, cDNA is captured on streptavidin beads which are placed in an oil emulsion mixture, creating separate water compartments containing only one template [89]. This partitioning minimizes hybridization between amplification products. Life Technologies has also introduced an isothermal template walking approach for library construction [90]. Bar-coded human exome fragment libraries are modified to contain 3′ poly-T overhangs. Solid-phase primers capture the nicked template DNA and are extended at room temperature with Bst polymerase. Next, isothermal strand displacement was run with Bst and a solution-phase primer. Compared to emPCR or bPCR, this template walking method does not use microbeads and is more cost-effective. Conversely, Illumina sequencing uses isothermal solid-phase bPCR. In this method, two oligos are situated on a glass slide. The template hybridizes with the first oligo, is elongated by a polymerase, and is washed away. The single-stranded sequence then bends over and hybridizes with a second oligo and a double-stranded bridge is created by the polymerase. The process is repeated until the cDNA has been sufficiently amplified [90].

Once a library is generated, sequencing may be performed. In the SOLiD platform, fluorescently labeled dinucleotide sequences hybridize with complementary template sequences and are ligated, and fluorescence is measured [91]. Illumina platforms utilize sequencing-by-synthesis, which involves adding fluorescently labeled nucleotides to a nucleotide chain [92]. After the addition of each nucleotide, the fluorescent signal is captured. Next, data analysis is performed, including sequence alignment to a reference genome, count generation, data pre-processing, and statistical analysis [93].

Compared to the preceding Sanger sequencing technology, NGS is not limited by the use of gel or polymer separation media and thus allows multiple samples to be run in parallel [94]. Given its ability to query the entire genome, NGS is ideal for novel biomarker discovery. As the cost continues to decrease, NGS is quickly becoming the preferred genome-wide quantification method [95–97]. However, implementing such technology in the clinic poses serious challenges. Like microarray, NGS data analysis is complicated and not standardized [93]. Several studies have used NGS in blood samples in order to identify candidate miRNA for CRC screening [98–100], but these studies are often validated using another more targeted approach (e.g., qPCR).

### Novel miRNA quantification tools

#### Isothermal amplification

All of the aforementioned methods must amplify genetic material in order to detect signal. While NGS employs isothermal bPCR and template walking, qPCR and microarray traditionally utilize thermocycling to create cDNA. This involves a denaturation step followed by several rounds of primer annealing and elongation by a Taq polymerase. By contrast, isothermal reactions are performed at a constant temperature. Several variations of isothermal amplification exist, including exponential amplification reactions, loop-mediated amplification, rolling circle amplification, duplex-specific nuclease signal amplification, and hybridization chain reaction [101].

**Fig. 1:**
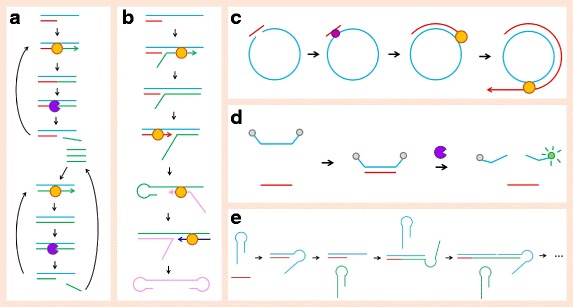
Summary of isothermal amplification techniques. **a** Exponential amplification using a nicking enzyme. **b** Exponential amplification without nicking enzyme. **c** Rolling circle amplification. **d** Duplex-specific nuclease amplification. **e** Hybridization chain reaction

Exponential amplification reactions employ a nicking enzyme to catalyze miRNA amplification. Jia et al. used an amplification template with a nicking enzyme recognition sequence flanked by two identical sequences that are complementary to the target miRNA [102]. Once the miRNA of interest hybridizes with the amplification template, it is extended by Vent (exo-) DNA polymerase, then cleaved by the nicking enzyme (Fig. 1a). The released product then serves as the trigger for the next amplification reaction. Using a SYBR Green dye, the product can be detected in real time. This procedure has been further modified using stem-looped amplification template and strand displacement, which eliminated the need for a nicking enzyme (Fig. 1b) [103]. In this design, an amplification template, forward inner primer, backward inner primer, and outer primer are required. At the 5′ end, the template contains a binding region for the outer primer, and at the 3′ end of the template, there is a sequence complementary to the target miRNA. In between the two ends, there are binding regions for the backward and forward inner primers. First, the forward inner primer binds the amplification template and is extended by Bst DNA polymerase. Next, the miRNA binds and is extended to displace the forward inner primer, creating a 5′ stem-loop from the forward inner primer. At the 3′ end of the stem-loop forward inner primer, the backward inner primer is elongated with Bst DNA polymerase and displaced by the backward outer primer, allowing the formation of the 5′ stem loop. SYBR Green can again be used to quantify the amplification product. These methods have been shown to have extremely high miRNA detection sensitivity, capturing signal at miRNA levels as low as 10^−10^ pmol [103]. However, primer design is complicated and if a nicking enzyme is used, the template must contain a recognition site.

Rolling circle amplification provides another isothermic technique for miRNA quantification. In this method, the miRNA of interest ligates with a circular ssDNA template and is extended with DNA polymerase (Fig. 1c). Once the full length of the template has been elongated, the product is displaced and the polymerase continues around the circular primer, creating many copies of the miRNA. This method was first used for miRNA by Jonstrup et al. in 2006 but has since been improved [104]. Modifications to the original method include the use of a hairpin DNA probe to trigger the rolling circle reaction [105, 106] and immediate miRNA quantification via an enzymatic luminescence assay [107] or a fluorescently labeled probe [108]. Solid-phase rolling circle amplification was also applied for NGS amplification by Drmanac et al. to avoid the need for precise template concentrations [109]. Rolling circle amplification is simple in that only one primer is necessary and may be optimized to perform either linear or exponential amplification. However, two enzymes are required, both a polymerase and a ligase, and the initial denaturation is not performed at room temperature.

Duplex-specific nuclease signal amplification employs a duplex-specific nuclease that specifically cleaves double-stranded DNA or DNA:RNA heteroduplexes. The use of duplex-specific nuclease signal amplification for miRNA detection was first introduced by Yin et al. [110]. In their design, a Taqman probe hybridizes with the miRNA of interest and the probe is digested by the duplex-specific nuclease, releasing a fluorescent signal (Fig. 1d). This method has been paired with Fe_3_O_4_@Ag nanoparticles [111], Au nanoparticles [112], and Au nanoparticles bound to MoS_2_ microcubes [113] for signal amplification. The duplex-specific nuclease is very effective at increasing assay specificity in that it selectively digests dsDNA and heteroduplexes but leaves ssDNA and imperfectly matched sequences untouched. However, the enzyme itself is isolated from the Kamchatka crab and is currently not widely available. The technique also results in linear, rather than exponential amplification which is not ideal for detection of weakly expressed miRNA.

Hybridization chain reaction is a method in which two DNA hairpin primers undergo a series of hybridization reactions to create a self-assembled DNA nanostructure (Fig. 1e) [114]. The reaction is initiated by a ssDNA that hybridizes with the sticky end of the first primer and opens the hairpin via strand displacement, creating a sticky end. That stick end opens the second hairpin primer and exposes a new sticky end that contains a sequence identical to the initiator, which can act on the first primer. This cycle continues, forming a nicked double helix. When a miRNA is used as the initiator, this method can be used for miRNA quantification and visualization. Signal detection has been facilitated through the use of Ag [115] and Au [116] nanoparticles, as well as fluorescently labeled primers that are stabilized by a tetrahedral DNA scaffold [117] or a graphene oxide surface [118]. Recently, Bi et al. coupled hybridization chain reaction with catalytic hairpin assembly, a method in which an initiator sequence catalyzes the self-assembly of hairpin primers to form complex nanostructures [119, 120]. Two DNA hairpins were used for catalytic assembly, and one unlabeled hairpin and one FAM-labeled hairpin were used for the hybridization chain reaction. When the miRNA of interest was introduced, it hybridized with the sticky end of the first primer and toehold-mediated strand displacement occurred as the first hairpin primer was opened. The exposed single-stranded sequence on the first primer then opened the second hairpin primer through an analogous reaction. The second primer then displaced the miRNA on the first primer, forming a branched DNA junction. Subsequently, the third and fourth hairpin primers catalyzed the hybridization chain reaction at each of the branches and the chemoluminescence signal was measured. The primary advantage of hybridization chain reaction is that it does not require a polymerase or any other enzyme, as it simply uses the potential energy of the hairpin primers. However, hybridization chain reaction only performs linear amplification and thus does not provide the same miRNA detection sensitivity as exponential amplification systems [114].

While isothermal amplification has not yet been used to quantify miRNA in CRC, it has been used to measure DNA and mRNA. Two investigations have used isothermal amplification to amplify DNA for microarray analysis of copy number variation. Cardoso et al. used Phi29 DNA polymerase and random hexamer primers for multiple strand displacement amplification with only 2 ng of DNA [121]. A subchromosomal 5q deletion was detected in tumor cells from patients with familial adenomatous polyposis (FAP). In another study, single primer isothermal amplification was used and a gain of 20q was found to be associated with tumor invasiveness in CRC patients [122]. Isothermal amplification of mRNA in CRC patients has been performed using loop-mediated exponential amplification [123, 124]. Higher levels of cytokeratin 19 (CK19) in lymph nodes was positively correlated with tumor size in stage I and II CRC patients.

The application of isothermal miRNA amplification in CRC screening has yet to be explored, but several other studies have highlighted the use of isothermal amplification in quantifying miRNA in other cancers. Persano et al. used a nicking enzyme amplification reaction to quantify higher levels of miR-10b in breast cancer cell lines and in serum of mice with breast tumors [125]. Catalytic hairpin assembly and rolling circle amplification were also used to detect high levels of miR-21 in breast cancer cell lines [126–128]. Additionally, rolling circle amplification was employed to measure high levels of miR-21 and miR-486-5p in lung cancer cell lines and in serum from lung cancer patients, respectively [106, 129]. Although isothermal amplification of miRNA is currently not a widespread methodology, it has tremendous potential to simplify screening across cancers.

#### Near-infrared technology

Traditional miRNA quantification tools require extensive sample preparation including miRNA isolation and amplification. While isothermal amplification has the potential to improve existing platforms by minimizing equipment demands and thus reducing cost, other technology has the potential to stand alone and possibly act as an alternative to commonly used techniques. Near-infrared (NIR) imaging for detection of miRNA in biological samples would potentially eliminate the need for PCR and complicated sample processing. Furthermore, it confers numerous advantages over its optical imaging counterparts, such as minimal photobleaching and autofluorescence [130].

NIR-emitting lanthanoids can be utilized as probes. For instance, ytterbium (III) is one of the 14 elements in the periodic table that belong to the lanthanoid series. Similar to other members in the series, ytterbium (III) ion is quite stable under physiological conditions and is environmentally benign. One unique property of ytterbium (III) ion is its narrow emission centered at 980 nm under illumination [131]. The emission is the result of an electronic transition from an inner *4f* shell; as a result, the emission wavelength is almost fixed and has little effect from the surrounding environment. Several other lanthanoid ions are also capable of emitting in the NIR region including the neodymium ion (1060 nm) and erbium ion (1540 nm); however, Zhong et al. have shown that ytterbium (III) outperforms others in terms of emission efficiency [132]. In the same experiment, ytterbium (III) was paired with a dye to further sensitize its emission. The dye molecule, upon excitation with a light source, transfers energy through its excited states (triplet or singlet states) to the ytterbium (III) ion. When the ytterbium (III) ion relaxes to its ground state, it produces characteristic emission. This process overcomes the requirement of a coherent and intense light source in direct excitation processes and is quite suitable and desirable for biomedical applications.

**Fig. 2:**
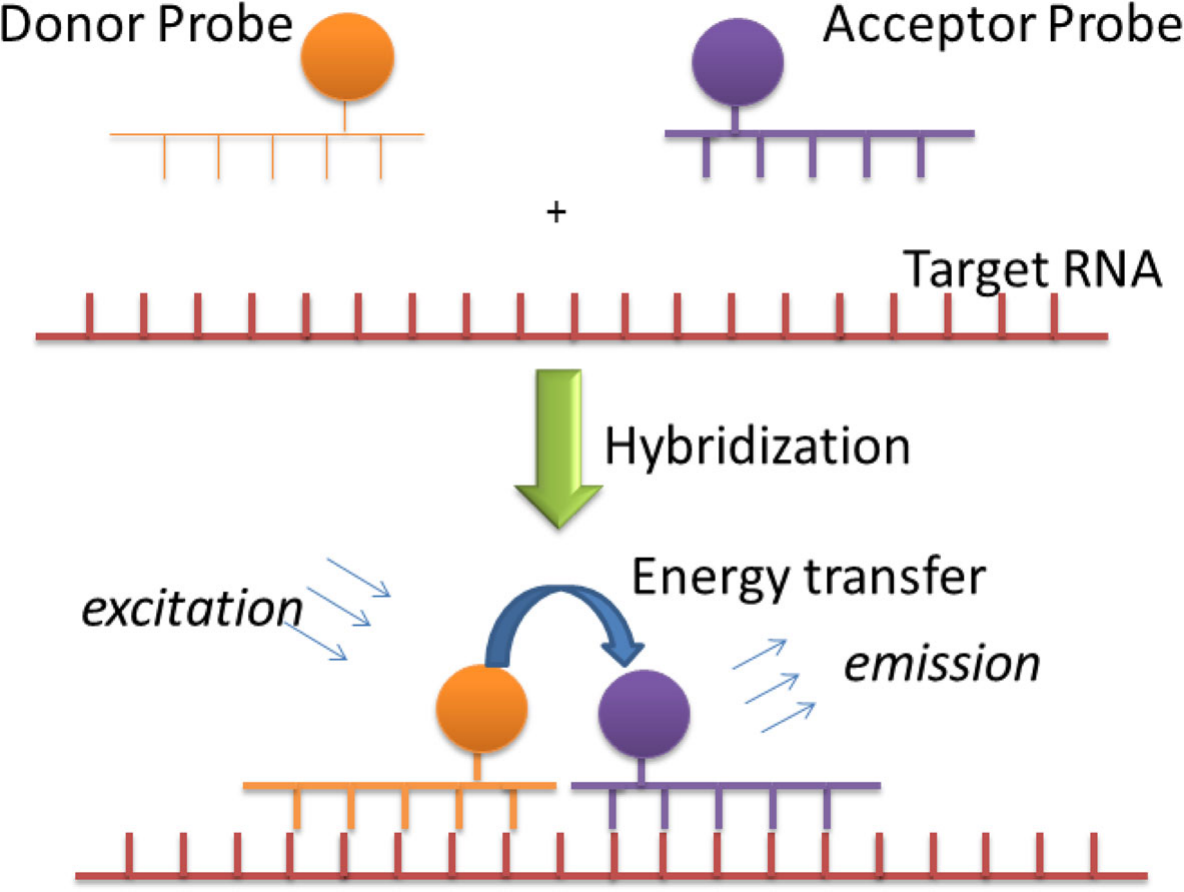
Working principle of linear FRET probes for nuclei acid detection. Two oligos are designed to base pair with the target RNA at adjacent sequences. One oligo is labeled with a donor chromophore at the 3′ end (Donor Probe). The other oligo is labeled with an acceptor chromophore at the 5′ end of the sequence (Acceptor Probe). Once the two oligos anneal to the target RNA, the chromophores are brought into proximity. Now, under light illumination, the donor probe will transfer energy to the acceptor probe and emit in NIR spectrum

It should be noted that sensitization can be achieved either through a Fӧrster resonance energy transfer (FRET) process or direct coordination of dye molecules to ytterbium (III) ion [133, 134]. Figure 2 illustrates the working principle of linear FRET probes using two antisense oligonucleotides. A donor chromophore and an acceptor chromophore are first labeled respectively on two oligonucleotides on the 3′ and 5′ ends. Upon mixing with a test sample, they hybridize with target nucleic acid in adjacent regions. This brings the donor and the acceptor into close proximity. Under light illumination, the donor transfers energy to the acceptor to produce fluorescence for quantification or imaging of target nucleic acid levels.

Visible light-emitting lanthanoids including europium (III) and terbium (III) have been reported for DNA, RNA, and avidin detection. Karhunen et al. separately attached a europium (III) chelate (an acceptor) and a dye molecule (a donor) to biotins [135, 136]. After mixing with avidin, the strong interaction between the avidin and the biotin brings the donor and the acceptor in proximity to initiate the energy transfer for emission under UV light excitation [135]. A similar homogeneous assay was also used to detect DNA in which a dye and europium (III) chelate are labeled separately on complementary oligonucleotides of target nucleic acids [136]. Once they hybridize with target DNA, the dye coordinates to the europium (III) ion and sensitizes its emission. Abe et al. linked a europium (III) (or terbium (III)) chelate and a 7-amino-4-methyl-2(1H)-quinolinone to different oligonucleotides [137]. Under illumination, the quinolinone was triggered by a luminogenic agent and converted to the quinoline to sensitize the europium (III) for emission. The luminogenic probe could detect DNA and RNA in a crude solution of living bacterial cells with a signal to noise ratio about 400. Two significant disadvantages were observed in these studies: (1) signal intensity was dramatically compromised due to the use of a time-gated technique to reduce the autofluorescence since europium (III) and terbium (III) both emit in the visible region (green and red) and (2) severe photobleaching due to the use of short wavelength light source (UV) because the dye molecules did not absorb long wavelength light. Both limitations are undesirable for nucleic acid detection.

Several studies have used lanthanoid-based probes for upconversion and detection in the visible spectrum, but only a few have harnessed the technology for nucleic acid detection at NIR wavelengths. Nonat et al. used ytterbium in a cyclen-ruthenium(phen)_3_ (phen: 1,10-phenanthroline) to bind DNA and measured changes in the NIR emission spectrum at different titrations of DNA [138]. This method was only employed to detect the presence of DNA rather than specific sequences. More recently, the transition metals ruthenium and osmium were used in heterometallic complexes with a pyrenyl-biimidazolate bridging ligand. It was again shown that the complex could act as a DNA intercalator for detection in the NIR spectrum [139]. NIR detection of miRNA has been accomplished through Ag_2_S quantum dots [140] and DNA-conjugated Ag nanoclusters [141]. However, these methods either required the use of an electrode or did not provide comparable miRNA detection sensitivity to amplification-based strategies, both of which would be problematic when rapid detection of less abundant miRNA is necessary.

Boron-dipyrromethene (BODIPY)-coupled lanthanide probes may be especially desirable for miRNA detection [132, 142, 143]. Zhong et al. synthesized a BODIPY ligand with 8-hydroxylquinoline in its *meso* position (8-HOQ-BODIPY) [132]. The ligand interacted with lanthanide ions, forming trisquinoline-like complexes that emitted in the NIR region upon visible light excitation. In a related experiment, the BODIPY dye was iodized with ytterbium (III) to form a probe with an empirical composition of [Yb(8-OQ–BODIPY–3I)_3_] [143]. The sensitization capability of the iodized [Yb(8-OQ–BODIPY–3I)_3_] probe was tested and it displayed an emission lifetime of ∼ 95 ± 17 μs. The emission efficiency was 4.75% (calculated from the equation *Φ*_Yb_ = *τ*_obs_/*τ*_0_, *τ*_0_ = 2 ms) [132, 144]. Under 543 nm visible light excitation, the complex exhibited maximum emission intensity at 975 nm. The results clearly demonstrate the ability and potential of BODIPY dyes to efficiently sensitize lanthanide ions for NIR emission.

Many optical probes are fluorescence-based molecules [145, 146],= and are widely used in a variety of medical diagnostic tests [147]. Several visible lights emitting lanthanoids including europium (III) and terbium (III) have been reported for DNA, RNA, and avidin detection [135–137]. The nucleic acid detection sensitivity of a probe relies upon the ratio of its fluorescent intensity and background signal. The significant overlap of biological substrate autofluorescence and fluorescence from the optical probes dramatically compromises detection sensitivity [148]. The NIR probe-based detection can overcome several drawbacks of current detection methods. First, it eliminates the autofluorescence. This is because nucleic acids only produce fluorescence in the visible region and do not produce emission in the NIR spectrum. By shifting the fluorescence of probes to the NIR region, the overlap that is quite significant in state-of-the-art probes will no longer exist. This creates “zero” background and increases the signal-to-noise (S/N) ratio of the detection dramatically [149]. Also, S/N benefits from weak Rayleigh scattering effect in the NIR region. Second, NIR can probes reduce photobleaching. Many commercially available probes require UV or near-UV light excitation, causing severe degradation of biological substrates. The proposed BODIPY-coupled NIR probes are efficiently excited by long wavelength light sources with much lower frequencies [143]. This prevents sample degradation due to photobleaching caused by higher energy light sources. Most importantly, the probes do not require tedious treatment of sample before and after the test. This not only expedites detection but also maintains the integrity of the sample for accurate detection of nucleic acids.

## Conclusion

CRC is unique in that both blood and fecal samples can be used for biomarker discovery. However, non-invasive screening for colorectal cancer is currently not as sensitive or specific as widespread endoscopic methods. Genetic tests may provide a more sensitive screening for at-risk individuals. Indeed, commercially available Cologuard® and Epi proColon® detect aberrant DNA methylation, and several studies have suggested that miRNA profiles in stool and in circulation also undergo drastic changes during CRC pathogenesis. With breakthroughs in basic science, technology must be developed to meet clinical demands. Current microarray, sequencing, and PCR-based methods of miRNA quantification are costly, complex, and have high time and resource demands. We suggest that isothermal amplification and NIR imaging may overcome the shortcomings of current techniques by reducing time and cost, eliminating the need for specialized equipment, and ensuring sensitive and specific miRNA detection. The development of novel technology will expedite biomarker discovery and enable their clinical implementation for effective CRC screening.

## Abbreviations

BODIPY: Boron-dipyrromethene
bPCR: Bridge PCR
CEA: Carcinoembryonic antigen
CRC: Colorectal cancer
CRT: Chemoradiation therapy
DNA: Deoxyribonucleic acid
emPCR: Emulsion PCR
FIT: Fecal immunochemical test
FOBT: Fecal occult blood test
FRET: Fӧrster resonance energy transfer
LNA: Locked nucleic acid
miR (miRNA): Micro ribonucleic acid
NGS: Next-generation sequencing
NIR: Near-infrared
PCR: Polymerase chain reaction
qPCR: Quantitative real time PCR
RNA: Ribonucleic acid
RT: Reverse transcription
SOLiD: Sequencing by oligonucleotide ligation and detection
